# *tAnGo*: a randomised phase III trial of gemcitabine in paclitaxel-containing, epirubicin/cyclophosphamide-based, adjuvant chemotherapy for early breast cancer: a prospective pulmonary, cardiac and hepatic function evaluation

**DOI:** 10.1038/sj.bjc.6604538

**Published:** 2008-07-29

**Authors:** A M Wardley, L Hiller, H C Howard, J A Dunn, A Bowman, R E Coleman, I N Fernando, D M Ritchie, H M Earl, C J Poole

**Affiliations:** 1 CR UK Department of Medical Oncology, Christie Hospital, Manchester M20 4BX, UK; 2 Warwick Medical School Clinical Trials Unit, University of Warwick, Coventry CV4 7AL, UK; 3 Cancer Research UK Clinical Trials Unit, University of Birmingham, Birmingham B15 2TT, UK; 4 Edinburgh Cancer Centre, Western General Hospital, Edinburgh EH4 2XU, UK; 5 Cancer Research Centre, Weston Park Hospital, Sheffield S10 2SJ, UK; 6 Cancer Centre, Queen Elizabeth Hospital, Birmingham B15 2TH, UK; 7 Beatson Oncology Centre, Western Infirmary, Glasgow G11 6NT, UK; 8 Oncology Centre, Addenbrookes Hospital, University of Cambridge, Cambridge CB2 0QQ, UK

**Keywords:** breast cancer, adjuvant chemotherapy, pulmonary, toxicity

## Abstract

tAnGo is a large randomised trial assessing the addition of gemcitabine(G) to paclitaxel(T), following epirubicin(E) and cyclophosphamide(C) in women with invasive higher risk early breast cancer. To assess the safety and tolerability of adding G, a detailed safety substudy was undertaken. A total of 135 patients had cardiac, pulmonary and hepatic function assessed at (i) randomisation, (ii) mid-chemotherapy, (iii) immediately post-chemotherapy and (iv) 6 months post-chemotherapy. Skin toxicity was assessed during radiotherapy. No differences were detected in FEV_1_ or FVC levels between treatment arms or time points. Diffusion capacity (TL_CO_) reduced during treatment (*P*<0.0001), with a significantly lower drop in EC-GT patients (*P*=0.02). Most of the reduction occurred during EC and recovered by 6-months post treatment. There was no difference in cardiac function between treatment arms. Only 11 patients had echocardiography/MUGA results change from normal to abnormal during treatment, with only five having LVEF<50%. Transient transaminitis occurred in both treatment arms with significantly more in EC-GT patients post-chemotherapy (AST *P*=0.03, ALT *P*=0.003), although the majority was low grade. There was no correlation between transaminitis and other toxicities. Both treatment regimens reported temporary reductions in pulmonary functions and transient transaminitis levels. Despite these being greater with EC-GT, both regimens appear well tolerated.

Breast cancer deaths in the UK have declined 20–30% since the late 1980s, despite an increasing incidence (41 720 new cases diagnosed in 2002). Increased use and improvement in systemic adjuvant therapy have undoubtedly contributed to this reduced mortality in the face of increasing incidence.

Combination chemotherapy has been shown to reduce recurrence rates when given after surgery to women at risk of relapse. Anthracycline-based regimens are the standard of care throughout the developed world reducing the breast cancer death rate ratios by 26–45% for younger and 17–24% for older women (EBCTCG, 2005). Modern anthracycline regimens appear to offer greater improvement (∼30% reduction in hazard ratio) over CMF (Levine *et al*, 2005; Poole *et al*, 2006a). Block sequential chemotherapy regimens have become standard in breast cancer after the demonstration that sequence and timing were important (Bonadonna *et al*, 2004).

The 1990s saw the initiation of many large randomised controlled trials of taxanes in the adjuvant therapy of breast cancer. Results from four first generation taxane trials are available and have resulted in licensing of taxanes for the adjuvant treatment of node-positive breast cancer. The addition of four cycles of paclitaxel after four cycles of doxorubicin and cyclophosphamide reduces the risk of recurrence by 17%, with manageable toxicity (Henderson *et al*, 2003; Mamounas *et al*, 2003), and became standard of care in the US in the late 1990s, for higher risk node-positive patients.

Based on pre-clinical evidence of a potentially favourable interaction between paclitaxel and gemcitabine (Kroep *et al*, 1999), as well as encouraging activity for gemcitabine in advanced breast cancer (Carmichael *et al*, 1995; Blackstein *et al*, 1997; Spielmann *et al*, 1997; Sanchez *et al*, 1998; Akrivakis *et al*, 1999; Colomer *et al*, 2000), the tAnGo trial was initiated in the year 2000 to test the addition of gemcitabine to block sequential anthracycline and paclitaxel adjuvant chemotherapy. The trial compared EC-GT (four cycles of epirubicin 90 mg m^−2^ and cyclophosphamide 600 mg m^−2^ day 1 every (q) 3 weeks, followed by four cycles of paclitaxel 175 mg m^−2^ every 3 h infusion day 1 and gemcitabine 1250 mg m^−2^ days 1 and 8 q3 weeks) with EC-T. The primary end point for the 3000 patient trial was disease-free survival (DFS), aiming to prove a 5% improvement from 70% 5-year DFS.

In view of the unknown acute and long-term sequelae of gemcitabine in combination with paclitaxel, radiation-sensitisation properties of gemcitabine (McGinn *et al*, 1996) and concern about the possibility of severe and/or delayed pulmonary (Gupta *et al*, 2002; Trodella *et al*, 2002; Maas *et al*, 2003) and hepatic toxicity (Poole *et al*, 2006b), a detailed toxicity and tolerability surveillance study was undertaken on an initial cohort of patients randomised into tAnGo. Hepatic, cardiac and pulmonary function and all adverse events (AEs) were monitored.

## Patients and methods

All patients randomised into tAnGo were eligible for this detailed safety substudy (DSS) until the accrual target had been met. Patients underwent tests at four time points: (i) randomisation; (ii) mid-chemotherapy (between cycles 4 and 5); (iii) immediately post-chemotherapy and (iv) 6-months post-chemotherapy. Late toxicity assessment included follow-up tests in the event of any symptomatic deterioration, and also at 5 and 10 years post-treatment. Results from the first four planned time points are presented here.

Pulmonary function assessments comprised spirometry tests, Forced Expiratory Volume in one second (FEV_1_, litres) as a measure of airway obstruction and Forced Vital Capacity (FVC, litres), single breath diffusion tests, measuring total lung capacity (TL_CO_, mmol min^−1^ kPa^−1^) (locally corrected for Hb), and gas diffusion of CO within lungs (K_CO_, mmol min^−1^ kPa^−1^ l^−1^). Cardiac assessments comprised ECG tests and either echocardiograms or MUGA (multi-gated acquisition) scans. Chest X-rays (CXR) were also undertaken, as were hepatic function tests serum AST and ALT. Radiotherapy acute skin toxicity treatment was collected weekly.

This trial was approved by Multi-Research Ethics Committee and conducted in accordance with the declaration of Helsinki.

## Statistical methods

Symptomatic pulmonary toxicity associated with single-agent gemcitabine was estimated to be 1.6% (Sara *et al*, 2000). Assuming a 1.5–2% incidence of symptomatic pulmonary toxicity, the accrual of 65 patients from each treatment arm into the tAnGo DSS would allow detection of a 10-fold difference in the risk of symptomatic pulmonary toxicity relative to this, with an 80% power at the 2-sided 5% level of significance.

FEV_1_ levels were categorised into normal, mild, moderate or severe obstruction using British Thoracic Society's COPD guidelines. ECGs, echocardiograms, MUGA scans and CXRs were classed as normal or abnormal by clinicians. Hepatic function was graded according to CTC toxicity criteria (version 2). Radiotherapy acute skin toxicities were categorised by clinicians into nil, mild, moderate or severe. Assessment times and treatment arms were then compared using *χ*^2^ tests, Fisher's exact tests or generalised linear models where appropriate.

Random effects modelling was applied to FVC and TL_CO_ levels over time, and results presented graphically as patients' raw scores, percentages of patients' baseline measures and the average patient values over time for each treatment arm as predicted by the model. Results were also categorised into low, normal or high levels (Quanjer, 1993).

## Results

A total of 135 tAnGo patients (69 randomised to EC-GT, 66 to EC-T) were entered into the DSS between August 2001 and October 2002. The DSS subgroup appeared balanced in terms of randomised treatment and also balanced across randomised treatment groups in terms of prognostic variables (Table 1). However, when comparing the DSS group with other patients randomised into tAnGo, the DSS group reported a smaller proportion of ER and PgR positive tumours, a larger proportion of HER2 positive tumours, higher nodal status, higher mastectomy rates and worse tumour grades. These expected differences reflect the change in tAnGo eligibility criteria, from ER-poor to any hormone status, which was implemented after the first 550 patients had been randomised, after the DSS had completed recruitment. DSS patients appear representative of breast cancer patients at large.

All 135 patients completed baseline assessments, with 117 (87%) being assessed at all four time points. The completeness of individual tests at each assessment time is generally high, except for hepatic function tests, which were often overlooked, especially at later assessments (Table 2).

### Pulmonary function results

**Figure 1:**
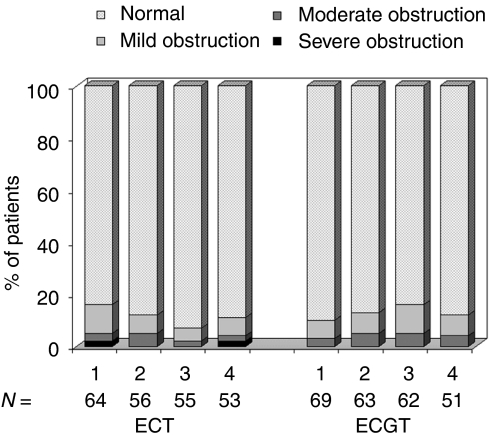
FEV_1_ results over the four time points.

Overall 88% of all FEV_1_ results were classed as normal (Figure 1). There was no indication in either treatment arm of a significant time effect on normal FEV_1_ levels (*P*>0.57), nor any differences detected between treatments at each of the four time points (*P*>0.23).

**Figure 2:**
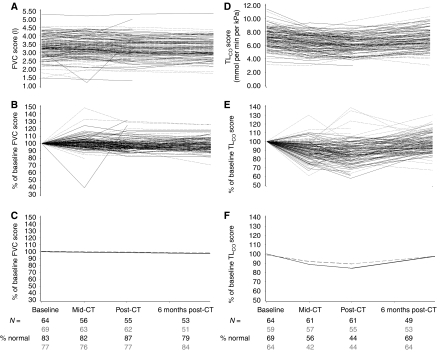
Pulmonary function levels over time (GREY dashed=EC-T patients, BLACK solid=EC-GT patients). (**A**) FVC levels. (**B**) FVC levels as percent of patient baseline level. (**C**) Average FVC levels over time for each treatment arm, predicted by random effects model. (**D**) TL_CO_ levels. (**E**) TL_CO_ levels as percent of patient baseline level. (**F**) Average TL_CO_ levels over time for each treatment arm, predicted by random effects model.

Eighty one percent of all FVC results were classed as normal (Figure 2A–C). In terms of the actual scores of the patients, there was no significant linear change over time (*P*=0.55) and no difference between treatments in terms of their change over time (*P*=0.84). Individually for the treatment arms, the populations' FVC scores, predicted from linear modelling, highlight the lack of differences over time and between treatments (Figure 2C).

A total of 56% of all TL_CO_ results were classed as normal (Figure 2D–F). Individual patient scores show a slight reduction in TL_CO_ during treatment, which appears slightly more pronounced in the EC-GT patients. The quadratic change over time observed (*P*<0.0001) suggests that the reduced TL_CO_ results during treatment tend to recover 6-months post treatment. The populations' TL_CO_ scores, predicted from quadratic modelling, show a significantly lower drop in TL_CO_ levels for EC-GT patients (*P*=0.02) (Figure 2F).

Dyspnoea was recorded as an AE during treatment in 32% of patients (43 of 135) and 9% of all cycles. Dyspnoea was CTC grade 2 in 89% and grade 3 in 4% of abnormal cycles. It was ungraded in the remaining 7%.

One hundred and twenty-two of the 135 DSS patients (90%) received radiotherapy, 13 (10%) did not. 134 of the 135 DSS patients (99%) have calculable chemotherapy course dose intensity. Adjusting the analysis of the pulmonary function tests by whether patients had received radiotherapy or not, and also by their chemotherapy dose intensity did not affect findings.

### Cardiac function

Overall 370 ECG, 323 echocardiogram and 168 MUGA results have been reported. Two patients switched mode of assessment of LVEF from initial to subsequent assessments: one from MUGA to echo and one vice versa – neither of these patients had any suggestion of cardiac dysfunction therefore they have been included in the analysis.

**Figure 3:**
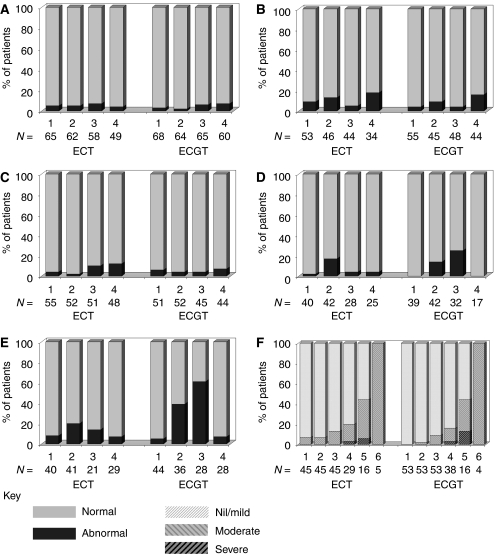
Pulmonary, Cardiac, Chest and Hepatic tests over four time points. Weekly RT toxicity. (**A**) ECHO/MUGA. (**B**) ECG. (**C**) Chest X-ray. (**D**) AST. (**E**) ALT. (**F**) Radiotherapy acute skin toxicity.

The majority of patients have normal echocardiograms/MUGAs (96% of patients at baseline, 97% at mid and 93% at end of chemotherapy, 94% at 6 months) and ECG's (94% of patients at baseline, 89% at mid and 96% at end of chemotherapy, 82% at 6 months (Figure 3A and B).

In 11 (8%) patients (6 EC-T; 5 EC-GT), ECHO/MUGA's were categorised as having changed from normal to abnormal throughout treatment. In two patients (assessed by echocardiography) LVEF value was not recorded. Of the remaining nine patients, two increased LVEF by 2%. For the other seven, the mean reduction in LVEF was 9% (range: 2–16%), with five patients having LVEF below 50%. Four patients had a fall of LVEF >10%; in three of these to <50%. The first abnormal test was the second scan in three patients, the third in six patients and the fourth in two patients. Three cardiac SAE's were reported from two patients: one patient experienced tachycardia mid-chemotherapy, the other asymptomatic reduction in LVEF (54–42%) mid-chemotherapy and symptomatic CHF 1 month later. Post-chemotherapy, LVEF returned to normal after this patient was treated with the ACE inhibitor lisinopril. In 6 of 11 patients, cardiac function testing recovered to normal at 6-month post-chemotherapy. There was no significant change in ECHO/MUGA cardiac function over time in either treatment arm (*P*>0.41) and no difference between treatments at any time point (*P*>0.36).

There was no difference in ECG results between treatments at any time point (*P*>0.27) and no difference within either treatment arm over time (*P*>0.12). The majority of the 34 possibly abnormal ECG's were minor ST-T wave changes (8), sinus brady/tachycardia (6), left bundle branch block (3) and left atrial abnormality (2). The ECG changed from normal to abnormal during treatment in only 15 patients (8 EC-T and 7 EC-GT). Only two of these had ECG changes suggestive of ischaemia, the others being minor ST-T or rhythm changes. Only one of these patients was deemed to have abnormal LVEF (55% on the first two echocardiograms to 57% on the third, which was deemed to be abnormal and 52% on the fourth also abnormal). These data illustrate that ECG's alone are not a very useful tool for assessing safety of chemotherapy.

At each time point, the majority of CXRs were classed as normal (Figure 3C). At each time point, no differences were detected between treatments (*P*>0.44). There was also no indication in either treatment arm of a time effect on CXRs (*P*>0.11). In all 14 patients (9 EC-T; 5 EC-GT) developed abnormal CXRs. In three, the CXR change was possible cardiomegaly/cardiac failure. Only one of these patients had a corresponding change in LVEF (increasing from 60% at baseline to 81%!) In two patients, changes were because of infection, one of these with a reported suggestion of pulmonary fibrosis. The reported possible pulmonary fibrosis at 6 months post-chemotherapy was, 2 months later, confirmed as pulmonary metastases. The second patient with a reported possible infection had reduced pulmonary function at the time of the abnormal CXRs.

### Hepatic function results

Overall 99% of baseline AST results and 94% of baseline ALT results were normal (Figure 3D and E). Subsequent time points showed normal rates drop slightly to 88% for AST and 75% for ALT. Abnormal results were: grade 1=42%, 2=50% 3=8% for AST and grade 1=64%, 2=18% and 3=18% for ALT. At three time points, no treatment differences were detected (*P*>0.99 AST, *P*>0.10 ALT). A higher abnormal rate was detected in EC-GT patients at post-chemotherapy (*P*=0.03 AST, *P*=0.003 ALT). There was no change over time in EC-T patients (*P*=0.09 AST, *P*=0.32 ALT) but a significant time effect in EC-GT patients (*P*=0.001 AST, *P*<0.0001 ALT) highlighting more abnormal levels at mid- and post-chemotherapy time points.

AST became abnormal in 15 patients (six EC-T, nine EC-GT) throughout treatment. In 13 patients with abnormal AST at mid-chemotherapy, none required dose reductions and only two dose delays (one in cycle 5 (reason not stated) and one in cycle 5 (for myelosuppression/neutropenia and oral/GI tract toxicity) and cycle 6 (for infection)) during the second half of their treatment regimen. All bar one patient received all their eight chemotherapy cycles (one only receiving five cycles because of an allergic reaction).

In 28 patients (8 EC-T, 20 EC-GT) ALT became abnormal during treatment. 22 of these were at mid-chemotherapy. Seven had dose reductions in the second half of their chemotherapy (13 cycles in total). Main reasons were neuropathy, arthralgia and myalgia (most commonly the latter two stated together). Three patients had dose delays in the second half of their chemotherapy (three cycles in total). The reasons were myelosupression/neutropenia, fatigue and a not stated reason. All 22 patients received all their eight chemotherapy cycles.

There was no association between elevated liver enzymes and abnormal FEV, FVC or TL_CO_ levels, nor ECHO/MUGA, ECG or CXR results.

### Acute skin toxicity results

One hundred twenty-two of the 135 patients (92%) received radiotherapy. In 98 (83%) (45 EC-T, 53 EC-GT), weekly acute skin toxicity was recorded. Toxicity was nil/mild in 87%, moderate in 12% and severe in 1%. Skin toxicity increased with time in each treatment arm (both *P*<0.0001, Figure 3F), but there was no difference between the chemotherapy groups at any time (*P*>0.33).

### Deaths

With a median follow-up of 48 months, 25 of 135 patients have died (19%). None were attributable to tAnGo treatment.

## Discussion

The tAnGo trial addresses the addition of gemcitabine to a block sequential anthracycline-taxane regimen for improvement of DFS. Case reports and retrospective series describing a potential problem of pulmonary toxicity from gemcitabine, particularly in proximity to radiotherapy, led us to undertake a systematic prospective quantitative substudy to assess pulmonary function by FEV_1_/FVC measurement, and carbon monoxide diffusion tests. Only TL_CO_ was significantly affected by chemotherapy, with a small reduction in both treatment arms, and more so with EC-GT. The majority of this effect occurred during the EC chemotherapy. Six months after treatment, TL_CO_ had recovered in both arms.

The effect of anthracycline and cyclophosphamide on pulmonary function has not been well documented until now. A significant reduction in DL_CO_ (see Appendix) has been demonstrated in patients with lung cancer following carboplatin/gemcitabine (Maas *et al*, 2003) or carboplatin/paclitaxel (Dimopoulou *et al*, 2002). In the tAnGo DSS, we saw a significantly greater drop in TL_CO_ levels for EC-GT patients than EC-T patients (*P*=0.02) but most of this difference had emerged during the EC phase of treatment. By the end of chemotherapy though, equal proportions of patients on the treatment arms were classed as normal, and recovery for all patients appeared complete by 6 months post-chemotherapy. There was no increase in reported pulmonary AEs. A third of the tAnGo DSS patients had dyspnoea recorded as an AE, in 9% of cycles. The majority was CTC grade 2.

The rate of cardiac dysfunction in this substudy was comparable with that in CALGB9344 (Henderson *et al*, 2003) where clinically important cardiac dysrhythmias, congestive heart failure, changes in LVEFs, or any other heart symptoms regardless of any possible relationship to treatment occurred in up to 2% of patients during treatment and was not significantly different between those who did and those who did not receive paclitaxel. Congestive heart failure was observed during protocol therapy in four (<1%) and six (<1%) patients and during post-treatment follow-up in 23 (1%) and 27 (2%) patients randomly assigned to CA (four cycles of cyclophosphamide 600 mg m^−2^ plus doxorubicin 60, 75 or 90 mg m^−2^) alone and CA plus paclitaxel (175 mg m^−2^), respectively. In the NSABP-B28 trial (four cycles of cyclophosphamide 600 mg m^−2^ plus doxorubicin 60 mg m^−2^ followed by four cycles paclitaxel 225 mg m^−2^ or no paclitaxel) the incidence of grade 3 or higher cardiac dysfunction either during or subsequent to therapy was 1.0% in the control and 0.9% in the experimental arm (Mamounas *et al*, 2005). The variation in cardiac function in different institutions involved in the tAnGo DSS was a noticeable feature, as was the fact that some patients in whom echocardiography was recorded as abnormal were also shown to have increased LVEF. Although recording of LVEF gives some useful information, it must be remembered that, as with all tests, it has limitations and does not substitute for appropriate liaison with cardiologists when required.

Transient elevation of transaminases were seen in both treatment groups of the tAnGo DSS with significantly more abnormal levels in EC-GT patients post-chemotherapy (AST *P*=0.03, ALT *P*=0.003, respectively), although the majority of these were low grade. The effect of gemcitabine on transaminases is well described and is a dose limiting toxicity at higher doses of gemcitabine (Fossella *et al*, 1997). Liver enzymes returned to normal by 6 months post-chemotherapy in all bar one EC-T patient with an abnormal AST and four patients (2 EC-T, 2EC-GT) with abnormal ALT levels. The long-term sequelae of disturbances of transaminases are unknown. There was no correlation between increases in liver enzymes and other toxicities, nor any difference in received dose intensity between patients with and without elevated liver enzymes during treatment (median dose intensity 96 and 98% respectively, *P*=0.43).

Gemcitabine has been reported to give rise to a radiation recall phenomenon when used in doses of 600 m m^−2^ and higher (Jeter *et al*, 2002). However, the tAnGo DSS showed no exacerbation of radiotherapy-related skin toxicity in EC-GT patients when compared with EC-T patients.

The requirement for large adequately powered trials of adjuvant treatment in breast cancer precludes comprehensive testing of all patients for all potential side effects. The concept of detailed safety substudies is well established in endocrine therapy trials (Eastell *et al*, 2006; Coleman *et al*, 2007) and detailed toxicity monitoring was performed in the CALGB9344 trial in which complete blood counts were obtained two times weekly, and all toxicities of grade 2 or more were collected on the first 325 patients enrolled. After the Data and Safety Monitoring Board reviewed this data, only toxicities of grades 3 or higher were recorded (Henderson *et al*, 2003). In the 135 tAnGo DSS patients, there was good protocol compliance especially with respect to the less standard procedures of LVEF monitoring and pulmonary function testing, for which 97 and 91% of patients respectively had three or more of the required tests and 86 and 76% respectively had all required time points. Somewhat surprisingly what might be considered more routine testing of transaminases was achieved less frequently.

In conclusion, the tAnGo DSS was undertaken to monitor the introduction of gemcitabine into adjuvant chemotherapy regimen for breast cancer. Both tAnGo treatments appear equally well tolerated, only causing mild to modest reduction in pulmonary function, which recovered completely by 6 months. Gemcitabine caused increased transaminase abnormalities of no clinical significance. This DSS clearly demonstrates that the addition of Gemcitabine to paclitaxel after epirubicin and cyclophosphamide for treatment of early breast cancer is safe. The DSS provides a useful means of detailed safety assessment for the addition of new agents into adjuvant treatment.

## Figures and Tables

**Table 1:** Patient and tumour characteristics

	**ECGT (*n*=69)**		**ECT (*n*=66)**	
	***N***	**%**	***N***	**%**
*Age*				
⩽50 years old	38	55	36	55
>50 years old	31	45	30	45
				
*ER status*				
Negative	56	81	53	80
Weakly-positive	7	10	7	11
Positive	6	9	6	9
				
*PgR status*				
Negative	43	62	34	52
Weakly-positive	7	10	10	15
Positive	2	3	2	3
Unknown	17	25	20	30
				
*Nodal status*				
Negative	13	19	12	18
1–3 nodes positive	24	35	24	36
⩾4 nodes positive	32	46	30	46
				
*HER2 status*				
+++	11	16	9	14
Other (0, 1+, 2+)	17	25	14	21
Not measured	41	59	43	65
				
*ECOG performance status*				
0	64	93	63	95
1	3	4	3	5
2	2	3	0	0
				
*Menopausal status*				
Pre	33	48	27	41
Peri	5	7	5	8
Post	27	39	28	42
Hysterectomy	2	3	1	1
Unknown	2	3	5	8
				
*Definitive surgery*				
Mastectomy	45	65	42	64
Breast conserving surgery	24	35	24	36
				
*Definitive surgery to entry (days)*				
Median (IQR)	32 (24–42)		33 (21–39)	
Range	11–57		15–56	
				
*Tumour diameter*				
⩽2 cm	22	32	29	44
>2 and ⩽5 cm	36	52	32	48
>5 cm	9	13	4	6
Unknown	2	3	1	2
				
*Distance to closest radial margin*				
<1 mm	5	7	2	3
1 to <5 mm	10	15	15	23
5 to <10 mm	12	17	6	9
⩾10 mm	17	25	16	24
Unknown	25	36	27	41
				
*Tumour type* a				
Ductal/NST	63	91	61	92
Lobular	9	13	4	6
Tubular/Cribform	1	1	4	6
Medullary	1	1	1	1
Other	1	1	2	3
Unknown	1	1	0	0
				
*Tumour grade*				
1 – Well differentiated	0	0	1	2
2 – Moderately differentiated	6	9	5	7
3 – Poorly differentiated	63	91	59	89
Unknown	0	0	1	2
				
*Vascular/lymphatic invasion reported*				
Yes	48	70	36	55
No	21	30	30	45
				
*Axillary nodes involved*				
Median (IQR)	3 (1–6)		3 (1–9)	
Range	0–24		0–27	
				

a Some specimens have multiple types.

**Table 2:** Completeness of assessments

	***N* (%) of patients with test results**				
	**4 time points**	**⩾3 time points**	**⩾2 time points**	**⩾1 time point**	**No results**
**Tests**	**(*N*=117)**	**(*N*=128)**	**(*N*=133)**	**(*N*=135)**	**(*N*=135)**
FEV_1_	89 (76)	117 (91)	132 (99)	135 (100)	0 (0)
FVC	89 (76)	117 (91)	132 (99)	135 (100)	0 (0)
TL_CO_	85 (73)	109 (85)	130 (98)	135 (100)	0 (0)
ECHO/MUGA	101 (86)	124 (97)	132 (99)	134 (99)	1 (1)
ECG	45 (38)	85 (66)	114 (86)	125 (93)	10 (7)
CXR	60 (51)	93 (73)	117 (88)	128 (95)	7 (5)
AST	30 (26)	57 (45)	84 (63)	94 (70)	41 (30)
ALT	26 (22)	55 (43)	82 (62)	104 (77)	31 (23)

## Appendix

TL_CO_ (reported in ml min kPa^−1^) and DL_CO_ (reported in ml^−1^ min^−1^ mm Hg) measure the same function and are related by a conversion factor (TL_CO_=0.335 DL_CO_).
